# Sexual function and satisfaction in young women with breast cancer: a 5-year prospective study

**DOI:** 10.1093/jncics/pkae111

**Published:** 2024-11-06

**Authors:** Ana Ferrigno Guajardo, Bryan F Vaca-Cartagena, Fernanda Mesa-Chavez, Alejandra Platas, Alan Fonseca, Marlid Cruz-Ramos, Melina Miaja Avila, Ana Laura Rodriguez, Paula Cabrera-Galeana, Alejandro Mohar, Cynthia Villarreal-Garza

**Affiliations:** Department of Medicine, Yale University School of Medicine, New Haven, CT 06510, United States; Breast Cancer Center, Hospital Zambrano Hellion TecSalud, Tecnologico de Monterrey, San Pedro Garza Garcia 66260, Mexico; Breast Cancer Center, Hospital Zambrano Hellion TecSalud, Tecnologico de Monterrey, San Pedro Garza Garcia 66260, Mexico; Breast Medical Oncology Unit, Instituto Nacional de Cancerología, Mexico City 14080, Mexico; Médicos e Investigadores en la Lucha contra el Cáncer de Mama, Mexico City 03810, Mexico; Breast Medical Oncology Unit, Instituto Nacional de Cancerología, Mexico City 14080, Mexico; Consejo Nacional de Humanidades, Ciencias y Tecnologías (CONAHCYT), Instituto Nacional de Cancerología, Mexico City 14080, Mexico; Breast Cancer Center, Hospital Zambrano Hellion TecSalud, Tecnologico de Monterrey, San Pedro Garza Garcia 66260, Mexico; Médicos e Investigadores en la Lucha contra el Cáncer de Mama, Mexico City 03810, Mexico; Breast Medical Oncology Unit, Instituto Nacional de Cancerología, Mexico City 14080, Mexico; Unidad de Epidemioogia e Investigacion Biomedica en Cancer, Instituto Nacional de Cancerologia/Instituto de Investigaciones Biomedicas/UNAM, Mexico City 14080, Mexico; Breast Cancer Center, Hospital Zambrano Hellion TecSalud, Tecnologico de Monterrey, San Pedro Garza Garcia 66260, Mexico; Médicos e Investigadores en la Lucha contra el Cáncer de Mama, Mexico City 03810, Mexico

## Abstract

**Background:**

Young women with breast cancer (YWBC) face unique challenges that can affect their sexual health. This study aimed to identify factors associated with sexual activity, function, and satisfaction in YWBC up to 5 years postdiagnosis.

**Methods:**

We conducted a prospective cohort study of 474 women 40 years of age or younger diagnosed with nonmetastatic breast cancer in Mexico. Sexual function and satisfaction were assessed using the Female Sexual Function Index and the Sexual Satisfaction Inventory, respectively. Factors associated with sexual health outcomes were examined using mixed-effects models.

**Results:**

The prevalence of sexual dysfunction increased from 33.6% at baseline to 52.9% at 4-5 years postdiagnosis. Factors associated with worse sexual function included older age (mean predicted FSFI score = −1.35, *P* = .037), treatment-induced amenorrhea (−2.86, *P < *.001), depression (−4.11, *P < *.001), and anxiety (−2.13, *P < *.001). Lower sexual satisfaction was associated with lower educational attainment (mean predicted SSI score = −5.61, *P *= .002), being single (−6.41, *P *<* *.001), treatment-induced amenorrhea (−3.76, *P *=* *.004), bilateral oophorectomy (−8.21, *P *= .017), depression (−11.29, *P < *.001), and anxiety (−7.50, *P < *.001). Quality of life, body image, and systemic therapy side effects significantly affected both outcomes. Three distinct trajectories of sexual function were identified: high (62.2%), intermediate (24.3%), and markedly declining (13.5%). Four trajectories of sexual satisfaction were found, ranging from intermediate-to-high (57.3%) to progressively worsening (27.5%).

**Conclusion:**

Sexual dysfunction is prevalent and persistent among YWBC. Multiple biological, psychological, and social factors influence sexual health outcomes in this population. These findings highlight the importance of routine screening and tailored interventions to address the sexual health of YWBC throughout survivorship.

## Background

Breast cancer (BC) is the most common malignancy in women, with an estimated 2.3 million new cases diagnosed in 2022.^1^ Given enhanced screening strategies and treatment advances, the survival of patients with BC has substantially improved in recent decades.^2^ The growing population of survivors underscores the importance of evaluating the long-term detrimental effects on quality of life (QoL) associated with this diagnosis. Sexual function and satisfaction are critical components of QoL that have been identified as prevalent yet underaddressed issues among BC survivors.^3-7^

BC treatment can profoundly disrupt sexual functioning. Estrogen deprivation, either due to chemotherapy-induced ovarian function suppression or as a consequence of endocrine therapy (ET), can lead to vaginal dryness, decreased libido, and dyspareunia.^8^ Additionally, psychological stressors such as emotional distress, poor body image, and systemic treatment side effects like fatigue can further impair sexual interest, function, and satisfaction.^9-12^ The interplay between these physiological and psychological factors can have a significant and long-acting impact on sexual well-being. Current guidelines highlight that sexual complaints are common among BC survivors, with the most frequent cited issues being decreased libido (23%-64%), arousal or lubrication concerns (20%-48%), orgasmic concerns (16%-36%), and dyspareunia (35%-38%).^13^

Among the survivor population, young women with BC (YWBC), defined as those diagnosed at ≤40 years of age,^14^ are particularly susceptible to experiencing detrimental effects on sexual health. In contrast to older patients, YWBC confront distinct challenges, including abrupt ovarian function suppression, premature ovarian failure, and age-specific psychosocial stressors related to career development, family planning, and child-rearing responsibilities.^14-17^

Unlike other acute treatment side effects, sexual problems tend to persist chronically and may even worsen over time if unaddressed.^18^^,^^19^ Limited prospective data exist on the long-term changes of sexual function and satisfaction in YWBC, especially in non-Caucasian populations who might experience distinct patterns or magnitudes of sexual health outcomes due to unique influences of cultural attitudes toward sexuality, family dynamics, and health-care access. Determining the sexual health needs of younger survivors is crucial to optimize survivorship care and improve overall well-being for this vulnerable group. The aim of this study was to identify factors associated with sexual activity, function, and satisfaction of YWBC in Mexico.

## Methods

### Trial design

Joven & Fuerte (J&F) is a prospective cohort study evaluating QoL outcomes among YWBC.^20^^,^^21^ Patients were recruited from 3 cancer referral centers across Mexico between November 2014 and July 2021. Eligible patients for this analysis were women 40 years of age or younger with diagnosis of BC within 6 months of enrollment. Patients with metastatic disease were excluded because their distinct treatment goals and prognosis could confound the assessment of long-term sexual health outcomes compared with early-stage survivors.

The patients who consented to participate were asked to complete a series of questionnaires at enrollment (baseline), 6 months, 1 year, 2-3 years, and 4-5 years postdiagnosis.^20^^,^^21^ No monetary incentives were offered for participation. For patients with disease recurrence during follow-up, data were included until time of progression. The protocol was approved by the institutional review board of Instituto Nacional de Cancerologia.

### Study outcomes

Sociodemographic data including relationship status, educational attainment, and employment status were self-reported. Sexual activity was also self-reported, as a response to the question “In the past 4 weeks, have you engaged in any sexual activity? This includes, but is not limited to, penetrative intercourse, oral sex, any other activities intended for sexual pleasure with a partner.” Clinical data were obtained directly from the electronic medical record.

The Female Sexual Function Index (FSFI) was used to measure sexual function in patients who had engaged in sexual activity within 4 weeks of survey completion. This 19-item questionnaire evaluates overall level of sexual function as well as 6 subdomains: sexual desire, arousal, lubrication, orgasm, pain, and satisfaction.^22^^,^^23^ Each item is graded based on a 5-point Likert scale, with higher scores indicating better sexual functioning. A total score lower than 26.55 is suggestive of female sexual dysfunction.^22^

Sexual satisfaction was evaluated using the Sexual Satisfaction Inventory (SSI) in patients who had engaged in sexual activity within 4 weeks preceding the survey. This questionnaire consists of 29 items that are scored based on a 5-point Likert scale, with higher scores representing greater satisfaction. Patients scoring 110 or lower are considered to have low sexual satisfaction, 111-123 moderate, and at least 124 high.^24^

Additional patient-reported outcome measures collected included the European Organisation for Research and Treatment of Cancer QOL Questionnaire Core 30 (QLQ-C30) and BC-Specific QOL Questionnaire (QLQ-BR23)^25^ and the Hospital Anxiety and Depression Scale (HADS).^26^

### Statistical analysis

Descriptive statistics were used to summarize sociodemographic and clinical characteristics. Differences in categorical variables were assessed using χ^2^ tests, whereas continuous variables were compared using *t* tests.

Mixed-effects models were created to identify factors associated with sexual activity, FSFI score, and SSI score using the maximum likelihood estimation method. The variables of interest, time since diagnosis, and the interaction between these were included in each model as fixed effects, whereas individual identification number was included as random effect. For each outcome, a final multivariate model was created using factors found to be statistically significant, as well as those found to be associated with being sexually inactive to adjust for potential confounding factors.

To identify the trajectory patterns during the follow-up period, group-based trajectory analysis was undertaken. Linear, quadratic, and cubic models with 1-6 trajectory groups were sequentially created. The model with the best fit was selected using lower Akaike information criterion as an indicator for better fit. Furthermore, an average posterior probability of assignment exceeding 70% and sufficient sample size per group (each group consisting of at least 5% of total sample) were used to ensure goodness of fit. Multinomial regression models were then used to identify variables associated with group assignment using the best trajectory group as reference.

Analyses were conducted using STATA 18.0. and R using the “GBMT” package version 0.1.3. All statistical tests were 2-sided with a *P* value less than .05 considered statistically significant. No adjustment for multiple comparisons was made.

## Results

Of 590 patients enrolled in J&F, data from 474 were included. Reasons for exclusion were de novo metastatic disease (n = 57), no data regarding sexual function or satisfaction (n = 45), unknown disease recurrence status (n = 11), missing date of BC diagnosis (n = 2), and less than 6 months from diagnosis to enrollment (n = 1).

### Sociodemographic and clinical characteristics

Most patients included in this analysis were married or in a domestic partnership (65.3%), had completed high school education (67.6%), and were unemployed (61.4%). The majority had stage IIB disease or higher (59.7%) and hormone receptor (HR) positive HER2 negative (52.7%) disease. For treatment, 74.1% underwent total mastectomy, 88.8% received chemotherapy, 72.6% had radiotherapy, and 63.7% received ET (Table 1).

**Table 1.** Sociodemographic and clinical characteristics of the cohort (missing data not shown).

	N (%)
**Age at diagnosis (years)**	
21-25	22 (4.6%)
26-30	61 (12.9%)
31-35	152 (32.1%)
36-40	239 (50.4%)
**Country of birth**	–
Mexico	434 (97.8%)
Other	10 (2.3%)
**Education**	–
None	1 (0.2%)
Elementary	37 (8.1%)
Middle	110 (24.1%)
High school	86 (18.8%)
Technical college	44 (9.6%)
College	149 (32.6%)
Postgraduate	30 (6.6%)
**Occupation**	–
Full-time	71 (16.7%)
Part-time	84 (19.8%)
Unemployed	261 (61.4%)
Student	9 (2.1%)
**Marital status**	–
Single	115 (27.0%)
Married	192 (45.1%)
Domestic partnership	86 (20.2%)
Divorced	28 (6.6%)
Widowed	5 (1.2%)
**Monthly household income**	–
<2700 MXN	120 (30.1%)
2700-6799 MXN	138 (34.6%)
6800-11 599 MXN	71 (17.8%)
11 600-34 999 MXN	39 (9.8%)
35 000-84 999 MXN	21 (5.3%)
≥85 000 MXN	10 (2.5%)
**Ever pregnant**	–
Yes	344 (77.7%)
No	99 (22.4%)
**Health insurance**	–
Private	29 (6.9%)
Public	347 (82.8%)
None	43 (10.3%)
**Stage at diagnosis**	–
0	11 (2.3%)
I	48 (10.1%)
II	230 (48.5%)
III	185 (39.0%)
**Histological type**	–
Ductal	429 (90.7%)
Lobular	15 (3.2%)
Mixed/Other	29 (6.1%)
**Histological grade**	–
Low	40 (9.3%)
Intermediate	178 (41.6%)
High	210 (49.1%)
**Subtype**	–
HR+/HER2-	250 (52.7%)
HR+/HER2+	68 (14.4%)
HR-/HER2+	33 (7.0%)
TNBC	123 (26.0%)
**Mastectomy type**	–
Total	351 (74.1%)
Partial	116 (24.5%)
None	7 (1.5%)
**Axillary surgery type**	–
ALND (+/- SNB)	283 (59.7%)
SLNB only	184 (38.8%)
None	7 (1.5%)
**Chemotherapy**	–
Yes	421 (88.8%)
No	53 (11.2%)
**Radiotherapy**	–
Yes	344 (72.6%)
No	130 (27.4%)
**Hormone therapy**	–
TMX alone	187 (39.5%)
TMX + AI	14 (3.0%)
TMX ± AI + GnRHa	101 (21.3%)
None	172 (36.3%)
**Ovarian protection with GnRHa**	–
Yes	59 (12.5%)
No	415 (87.6%)
**Contralateral mastectomy**	–
Yes	81 (17.1%)
No	393 (82.9%)
**Oophorectomy**	–
Yes	37 (25.3%)
No	109 (74.7%)
**Breast reconstruction**	–
Immediate	148 (31.6%)
Delayed	37 (7.9%)
No	283 (60.5%)

Education levels: Elementary (grades 1-6), Middle (grades 7-9), High school (grades 10-12). Technical college offers vocational training, whereas college refers to university-level education.

1 USD ≈ 20 MXN (average during study period).

Abbreviations: MXN = Mexican peso; HR = hormone receptor; HER2 = human epidermal growth factor receptor 2; TNBC = triple-negative breast cancer; ALND = axillary lymph node dissection; SLNB = sentinel lymph node biopsy; TMX = tamoxifen; AI = aromatase inhibitor; GnRHa = gonadotropin-releasing hormone agonist.

### Sexual activity

**Figure 1.**
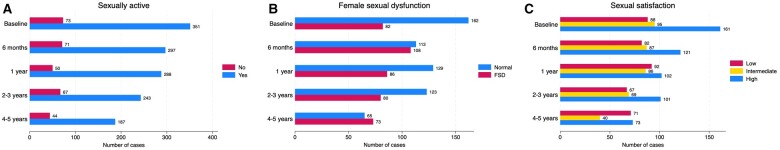
Number of patients sexually active (**A**), with an FSFI total score indicative of female sexual dysfunction (**B**), and different levels of sexual satisfaction as measured by SSI (**C**) at each timepoint assessed. Abbreviations: FSFI = Female Sexual Function Index; FSD = female sexual dysfunction; SSI = Sexual Satisfaction Inventory.

The proportion of sexually active patients was as follows: 82.8% at baseline, 80.7% at 6 months, 85.2% at 1 year, 78.4% at 2-3 years, and 81.0% at 4-5 years postdiagnosis (Figure 1).

**Figure 2.**
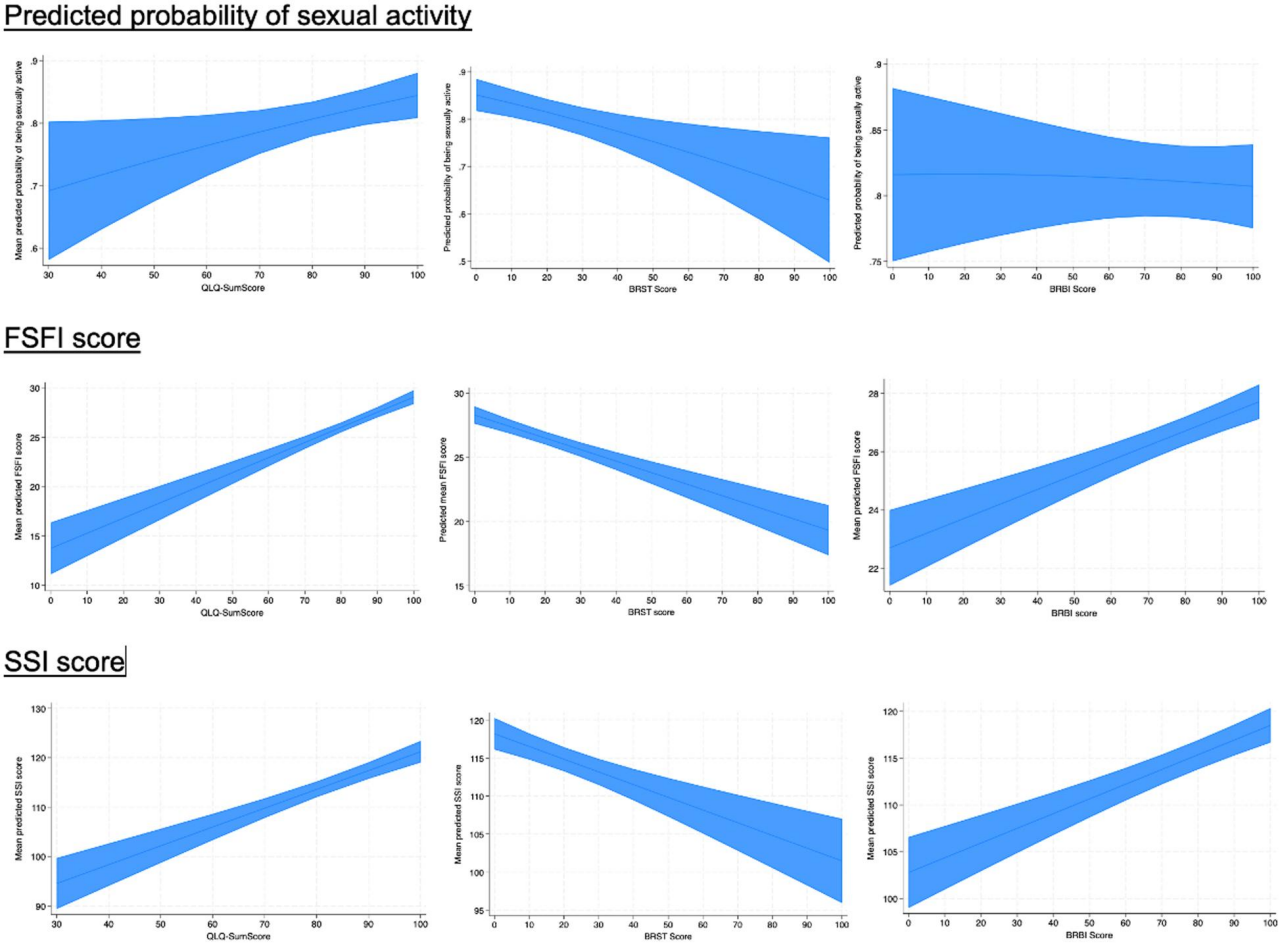
Predicted probability of being sexually inactive and mean FSFI and SSI scores according to QoL as measured by QLQ C30, and burden of systemic therapy side effects and body image as measured by the QLQ-BR23. Abbreviations: FSFI = Female Sexual Function Index; SSI = Sexual Satisfaction Inventory; QLQ-SumScore = QLQ-C30 summary score; BRST = score of the systemic therapy side effects symptom subscale of QLQ-BR23; BRBI = score of the body image functional subscale of QLQ-BR23.

The factors associated with a higher mean predicted probability of being sexually active were having an educational level of high school or higher (*P* =* *.004), being in a relationship (*P < *.001), and having children (*P* =* *.004). Furthermore, QoL directly correlated with probability of being sexually active (0.19%, 95% confidence interval [CI] = 0.03% to 0.35%, *P *=* *.015), whereas a higher burden of systemic therapy side effects was inversely correlated (−0.19%, 95% CI = −0.31% to −0.08%, *P *=* *.001) (Figure 2). In a multivariate model, the association of sexual activity with educational level (β = 0.85, *P = *.007) and relationship status (β = 2.89, *P < *.001) remained significant.

### Sexual function

**Figure 3.**
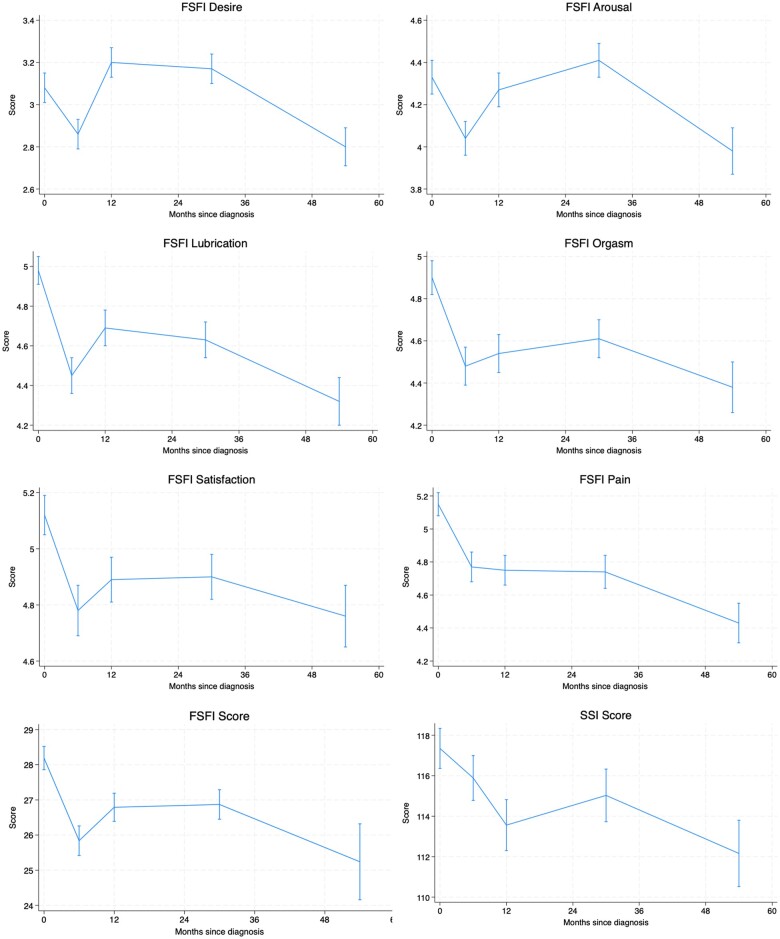
Mean scores with standard error at each follow-up. Abbreviations: FSFI = Female Sexual Function Index; SSI = Sexual Satisfaction Inventory.

Mean scores and standard error (SE) of each individual subscale of the FSFI questionnaire in sexually active patients are presented in Figure 3. Paired *t* tests showed a statistically significant decline in all FSFI subscales 6 months after BC diagnosis compared with baseline: desire (−0.26, SE = 0.08, *P = *.002), arousal (−0.29, SE = 0.11, *P = *.009), lubrication (−0.61, SE = 0.12, *P < *.001), orgasm (−0.55, SE = 0.11, *P < *.001), satisfaction (−0.4, SE = 0.12, *P = *.001), pain (−0.53, SE = 0.13, *P < *.001), and FSFI total score (−2.75, SE = 0.56, *P < *.001). At 4-5 years, the FSFI total score and its subscales remained numerically inferior to baseline, with *P* values signaling statistical significance for all but the arousal domain.

The prevalence of sexual dysfunction among sexually active patients was as follows: 33.6% at baseline, 48.9% at 6 months, 40.0% at 1 year, 39.4% at 2-3 years, and 52.9% at 4-5 years post-diagnosis (Figure 1).

**Figure 4.**
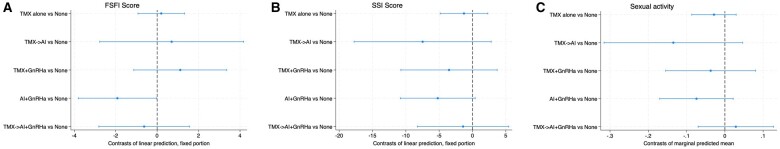
Contrasts of mean predicted Female Sexual Function Index score (**A**), Sexual Satisfaction Inventory score (**B**), and probability of being sexually inactive (**C**) by mixed models according to type of hormone therapy received.

Factors associated with worse sexual functioning were older age at diagnosis (*P = *.037), treatment-induced amenorrhea (*P *<* *.001), depression (*P *<* *.001), and anxiety (*P *<* *.001) (Table 2). Although receiving ET (ie, yes vs no) was not associated with a statistically significant change in FSFI score, patients who were treated with an aromatase inhibitor (AI) combined with a GnRH agonist (GnRHa) had statistically significant lower FSFI scores compared with those who did not receive ET (−1.92, *P = *.047) (Figure 4). Additionally, mean predicted FSFI scores directly correlated with QoL (+0.15, 95% CI = 0.12 to 0.18, *P *<* *.001) and body image (+0.05, 95% CI = 0.04 to 0.07, *P *<* *.001) and were inversely correlated with systemic therapy side effects (−0.09, 95% CI = −0.11 to −0.07, *P *<* *.001) (Figure 2). In a multivariate model, the association of sexual function with time (β = −0.04, *P *<* *.001), age >30 years (β = −1.49, *P *=* *.033), treatment-induced amenorrhea (β = −2.28, *P *<* *.001), overall QoL (β = 0.10, *P *<* *.001), and body image (β = 0.02, *P *=* *.017) remained statistically significant.

**Table 2.** Difference in mean predicted probability (95CI) of being sexually active and mean predicted total scores (95CI) of FSFI/SSI per variable studied using mixed-effects regression models.

Variable	Being sexually active		FSFI score		SSI score	
	Predicted probability	*P*	Predicted score	*P*	Predicted score	*P*
Age at diagnosis (> vs ≤30 years)	−0.05 (−0.11 to +0.02)	.139	**−1.35** **(−2.61 to −0.08)**	**.037**	−2.59 (−6.63 to +1.44)	.208
Educational attainment (≥ vs <high school)	**+0.09** **(+0.03 to +0.14)**	**.004**	+1.01 (−0.08 to +2.11)	.069	**+5.61** **(+2.14 to +9.09)**	**.002**
Occupation (full/part time/student vs unemployed)	+0.02 (−0.02 to +0.06)	.411	−0.08 (−0.87 to +0.70)	.832	+0.98 (−1.27 to +3.23)	.395
Relationship status (partnered vs no)	**+0.36** **(+0.30 to +0.43)**	**<.001**	+1.05 (−0.42 to +2.52)	.163	**+6.51** **(+3.27 to +9.76)**	**<.001**
Having children (yes vs no)	**+0.10** **(+0.03 to +0.17)**	**.004**	−0.46 (−1.78 to +0.86)	.493	−2.87 (−6.87 to +1.13)	.159
Stage at diagnosis (≥ vs <IIB)	−0.01 (−0.07 to +0.04)	.589	−0.26 (−1.27 to +0.75)	.614	+0.08 (−3.11 to +3.27)	.961
Hormone receptor status (positive vs negative)	−0.04 (−0.09 to +0.01)	.145	−0.28 (−1.34 to +0.77)	.596	−1.69 (−5.00 to +1.63)	.319
HER2 status (amplified vs no)	−0.00 (−0.06 to +0.06)	.940	−0.13 (−1.35 to +1.08)	.830	0.25 (−3.55 to +4.03)	.899
Mastectomy type (total vs partial)	0.01 (−0.05 to +0.07)	.705	0.08 (−1.07 to +1.22)	.896	−1.56 (−5.20 to +2.07)	.399
Axillary surgery type (ALND vs SLNB only/none)	−0.00 (−0.05 to +0.05)	.928	0.22 (−0.80 to 1.23)	.676	1.05 (−2.14 to +4.25)	.518
RT (yes vs no)	−0.01 (−0.06 to +0.05)	.816	−0.26 (−1.39 to +0.88)	.660	−1.27 (−4.79 to +2.25)	.479
Chemotherapy (yes vs no)	−0.02 (−0.10 to +0.05)	.532	−0.71 (−2.24 to +0.82)	.365	−1.12 (−6.03 to +3.80)	.655
ET (yes vs no)	−0.04 (−0.09 to +0.01)	.159	−0.09 (−1.13 to +0.94)	.861	−2.52 (−5.77 to +074)	.130
Anti-HER2 therapy (yes vs no)	−0.01 (−0.08 to +0.05)	.690	−0.25 (−1.50 to +0.99)	.688	0.50 (−3.35 to +4.36)	.798
Breast reconstruction (yes vs no)	+0.02 (−0.03 to +0.07)	.520	−0.39 (−1.42 to +0.63)	.450	−1.03 (−4.24 to +2.18)	.529
Contralateral mastectomy (yes vs no)	+0.04 (−0.02 to +0.11)	.184	−0.28 (−1.55 to +0.99)	.665	0.83 (−3.23 to +4.90)	.689
Bilateral oophorectomy (yes vs no)	+0.02 (−0.08 to +0.11)	.760	−0.17 (−2.18 to +1.84)	.867	**−8.21** **(−14.93 to −1.49)**	**.017**
Treatment-induced amenorrhea (yes vs no)	+0.02 (−0.03 to +0.06)	.431	**−2.86** **(−3.71 to −2.00)**	**<.001**	**−3.76** **(−6.29 to −1.23)**	**.004**
Depression (HADS-D ≥ vs <8)	−0.04 (−0.14 to +0.07)	.510	**−4.11** **(−6.10 to −2.12)**	**<.001**	**−11.29** **(−17.01 to −5.57)**	**<.001**
Anxiety (HADS-A ≥ vs <8)	−0.02 (−0.07 to +0.03)	.456	**−2.13** **(−3.19 to −1.06)**	**<.001**	**−7.50** **(−10.54 to −4.47)**	**<.001**

Values in bold are statically significant.

Abbreviations: 95CI = 95% confidence interval; FSFI = Female Sexual Function Index; SSI = Sexual Satisfaction Inventory; HER2 = human epidermal growth factor receptor 2; ALND = axillary lymph node dissection; SLNB = sentinel lymph node biopsy; RT = radiation therapy; ET = endocrine therapy; HADS = Hospital Anxiety and Depression Scale.

**Figure 5.**
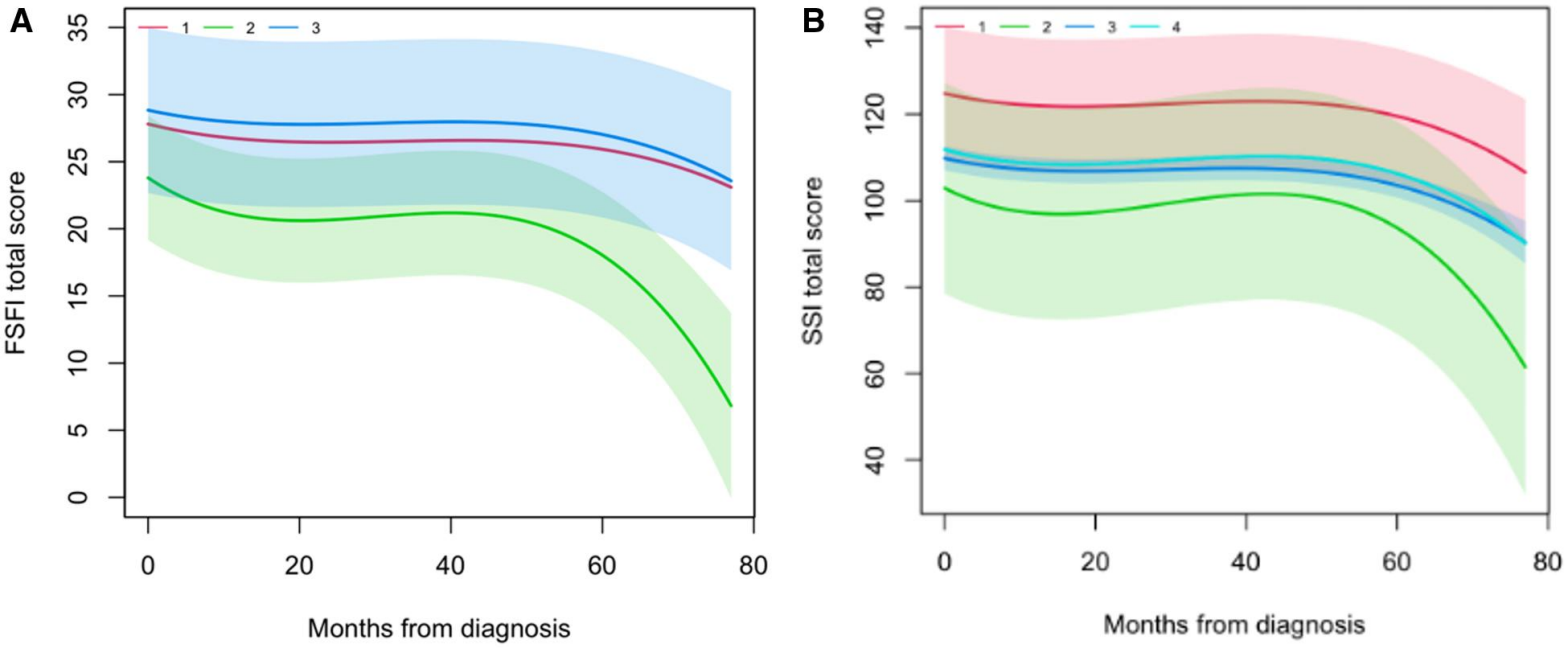
Distinct trajectories for sexual function (**A**) and sexual satisfaction (**B**) identified in the first 5 years postdiagnosis. In part A, 3 groups are identified: those with relatively stable Female Sexual Function Index (FSFI) score throughout follow-up (groups 1 and 3, **red** and **blue**) and those with marked worsening with longer follow-up (group 2, **green**). In part B, 4 groups are identified: those with worsening Sexual Satisfaction Inventory (SSI) score with longer follow-up (group 2, **green**), those with intermediate-to-high sexual satisfaction (group 4, light blue), those with low sexual satisfaction (group 3, **darkblue**), and those with high sexual satisfaction (group 1, **red**).

Group-based trajectory analysis showed 3 distinct sexual function trajectories: 62.2% of patients were classified as having high FSFI scores throughout the follow-up period, 24.3% had intermediate scores, and 13.5% experienced a marked decline in sexual function with time (Figure 5). Unadjusted regression models showed that depression (*P = *.001), anxiety (*P = *.005), overall QoL (*P = *.002), and body image (*P = *.002) were associated with sexual function group trajectory assignment (Table 3). These associations were not statistically significant in a multivariate model.

**Table 3.** Unadjusted relative risk ratios for assignment to worsening trajectory group with classification to the best trajectory group as reference.

Variable	Worsening female sexual function		Worsening sexual satisfaction		
	RRR (95CI)	*P*	RRR (95CI)		*P*
Age at diagnosis (≤ vs >30 years)	0.50 (0.24 to 1.05)	.068	0.65 (0.37 to 1.15)		.139
Educational attainment (≥ vs <high school)	0.79 (0.46 to 1.34)	.379	**0.49 (0.31 to 0.78)**		**.002**
Occupation (full/part time/student vs unemployed)	1.60 (0.94 to 2.73)	.085	0.88 (0.55 to 1.40)		.587
Monthly household income (≥ vs <6800 MXN)	0.77 (0.44 to 1.33)	.346	0.88 (0.55 to 1.42)		.606
Type of health insurance (private vs public/none)	1.91 (0.80 to 4.56)	.148	1.29 (0.55 to 3.02)		.554
Relationship status (partnered vs no)	1.23 (0.52 to 2.91)	.632	**0.46 (0.26 to 0.82)**		**.008**
Having children (yes vs no)	1.15 (0.59 to 2.23)	.686	**2.15 (1.18 to 3.92)**		**.012**
Stage at diagnosis (≥ vs <IIB)	0.95 (0.58 to 1.55)	.825	0.89 (0.58 to 1.35)		.576
Hormone receptor status (positive vs negative)	1.17 (0.70 to 1.97)	.550	1.02 (0.66 to 1.58)		.934
HER2 status (amplified vs no)	0.97 (0.52 to 1.78)	.910	1.09 (0.66 to 1.81)		.730
Mastectomy type (total vs partial)	1.48 (0.82 to 2.66)	.195	1.11 (0.69 to 1.80)		.664
Axillary surgery type (ALND vs SLNB only/none)	0.88 (0.53 to 1.44)	.599	**0.65 (0.43 to 0.99)**		**.046**
RT (yes vs no)	0.95 (0.55 to 1.66)	.868	1.27 (0.79 to 2.06)		.329
Chemotherapy (yes vs no)	1.19 (0.53 to 2.67)	.667	0.76 (0.40 to 1.44)		.395
Endocrine therapy (yes vs no)	1.02 (0.62 to 1.69)	.936	1.27 (0.82 to 1.96)		.286
Anti-HER2 therapy (yes vs no)	1.04 (0.56 to 1.93)	.898	0.97 (0.58 to 1.63)		.905
Breast reconstruction (yes vs no)	1.27 (0.77 to 2.09)	.356	1.12 (0.73 to 1.72)		.600
Contralateral mastectomy (yes vs no)	1.10 (0.58 to 2.06)	.778	1.07 (0.63 to 1.82)		.812
Bilateral oophorectomy (yes vs no)	1.58 (0.61 to 4.12)	.346	**3.81 (1.70 to 8.52)**		**.001**
Depressive symptom burden at baseline (HADS-D score)	**1.15 (1.06 to 1.25)**	**.001**	**1.18 (1.09 to 1.27)**		**<.001**
Anxiety symptom burden at baseline (HADS-A score)	**1.11 (1.03 to 1.20)**	**.005**	**1.13 (1.06 to 1.21)**		**<.001**
Overall QoL at baseline (QLQ-SumScore)	**0.96 (0.94 to 0.99)**	**.002**	**0.97 (0.95 to 0.99)**		**.001**
Body image at baseline	**0.98 (0.97 to 0.99)**	**.002**	**0.99 (0.98 to 1.00)**		**.003**

Abbreviations: RRR = relative risk ratio; 95CI = 95% confidence interval; MXN = Mexican peso; HER2 = human epidermal growth factor receptor 2; ALND = axillary lymph node dissection; SLNB = sentinel lymph node biopsy; RT = radiation therapy; HADS = Hospital Anxiety and Depression Scale; QoL = quality of life; QLQ-SumScore = European Organisation for Research and Treatment of Cancer QOL Questionnaire Core 30 summary score.

### Sexual satisfaction

Mean SSI score and SE in sexually active patients are presented in Figure 3. Paired *t* tests showed a statistically significant decline in sexual satisfaction (mean difference = −2.49, SE = 1.06, *P = *.020) that did not recover to baseline values at 4-5 years postdiagnosis. The prevalence of low sexual satisfaction was as follows: 25.6% at baseline, 28.3% at 6 months, 32.9% at 1 year, 28.2% at 2-3 years, and 38.6% at 4-5 years postdiagnosis.

Variables associated with better sexual satisfaction were higher educational attainment (*P* = .002), being in a relationship (*P *< .001), not undergoing bilateral oophorectomy (*P* = .017), not experiencing treatment-related amenorrhea (*P* = .004), and not having depression (*P *< .001) or anxiety (*P *< .001). Like FSFI scores, SSI scores directly correlated with QoL (predicted mean score = 0.38, 95% CI = 0.29 to 0.47, *P *<* *.001) and body image (0.16, 95% CI = 0.11 to 0.20, *P *<* *.001) and were inversely correlated with systemic therapy side effects (−0.17, 95% CI = −0.23 to −0.10, *P *<* *.001) (Figure 2). In a multivariate model, the association of sexual satisfaction with being in a relationship (β = 6.64, *P = *.047), bilateral oophorectomy (β = −8.42, *P *=* *.020), and anxiety (β = −7.79, *P = *.023) remained statistically significant.

Group-based trajectory analysis showed 4 distinct sexual satisfaction trajectories: 57.3% of patients were classified as having intermediate-to-high sexual satisfaction throughout the follow-up period, 7.8% had intermediate-to-low satisfaction, 7.4% had low satisfaction, and 27.5% experienced low satisfaction that worsened to very low as time elapsed from diagnosis (Figure 5). Unadjusted models showed that the variables associated with assignment to the worsening group trajectory were having children (*P *=* *.012) and bilateral oophorectomy (*P *=* *.001), whereas higher educational level (*P = *.002), being in a relationship (*P *=* *.008), and undergoing axillary lymph node dissection (*P *=* *.046) were protective. Similar to FSFI trajectories, higher depression and anxiety symptom burden at baseline were associated with assignment to the worsening satisfaction group (*P < *.001), whereas higher QoL and better body image at baseline were protective factors (*P *=* *.001 and *P *=* *.003, respectively) (Table 3). An adjusted multinomial model showed that the association with being in a relationship (relative risk ratio [RRR] = 0.19, *P *=* *.016) was the only factor that remained statistically significant.

## Discussion

This study is one of the largest cohorts characterizing the trajectory of sexual function and satisfaction in YWBC. Furthermore, it is the first study to longitudinally examine the sexual health outcomes of this population in Latin America. Our findings underscore the pervasive detrimental impact that BC exerts on multiple domains of sexuality in this vulnerable population.

In our cohort, 78%-85% of patients were sexually active in the first 5 years after BC diagnosis, which is similar to what has been reported in prior studies.^27^ The factor found to have the strongest association with sexual activity was relationship status, validating prior literature that highlights the importance of relationship-related variables for remaining sexually active after BC diagnosis.^28^

Prior literature has demonstrated that patients with cancer have a higher rate of sexual dysfunction compared with the general population.^29-31^ Wettergren et al.^29^ explored the prevalence of sexual dysfunction among women aged 18-39 years and found that 66% women with reproductive cancers reported sexual dysfunction in at least 1 domain compared with 53% of the participants from the general population (odds ratio = 1.69, 95% CI = 1.27 to 2.24). Similarly, a high level of female sexual dysfunction and dissatisfaction were identified throughout the follow-up period in our cohort. The fact that mean scores in most FSFI domains did not return to baseline after 5 years postdiagnosis is particularly notable because it highlights that sexual health issues are not only prevalent in this population, but also persistent. This chronic nature of sexual dysfunction and dissatisfaction aligns with the theory that BC treatment can induce abrupt and profound hormonal changes that precipitate sexual problems, which can then become self-reinforcing over years if not properly addressed. This is consistent with prior literature demonstrating that the proportion of BC survivors experiencing sexual issues remains stable or grows in the first years postdiagnosis, perhaps related to underutilization of supportive care strategies.^32-34^

We found that variables associated with sexual function included age, overall QoL, body image, and treatment-induced amenorrhea. Notably, reception of ET overall was not associated with worse sexual health outcomes, perhaps reflecting the possibility of chemotherapy-induced amenorrhea and use of GnRHa for ovarian protection in young patients with HR negative disease. However, among patients with hormone-sensitive tumors, the use of AI with GnRHa showed worse sexual function likely related to profound estrogen deprivation, leading to a higher burden of adverse effects.^35^ Similarly, variables associated with sexual satisfaction included relationship status, education, surgical oophorectomy, and anxiety, again highlighting the importance of emotional closeness with partners, abrupt estrogen deprivation, health literacy, and emotional distress for maintaining sexual health in this population.

This study provides novel granularity by delineating distinct trajectory groups, with more than one-tenth of patients experiencing a marked delayed deterioration in sexual function and approximately one-fourth experiencing progressively worsening sexual satisfaction, suggesting a delayed-onset pattern of sexual health problems in a subset of patients. Interestingly, anxiety and depression symptoms at baseline, as well as overall QoL and body image, were associated with being in the worsening sexual function group in unadjusted models, potentially reflecting a deeper influence of psychosocial factors than clinical characteristics. In contrast, variables associated with the worsening sexual satisfaction trajectory also included undergoing surgical oophorectomy and having children, whereas being in a relationship was a protective factor. The first might be secondary to surgically induced premature menopause leading to enhanced hormonal symptoms that can negatively affect sexual satisfaction. The protective effect of being in a relationship may be attributed to having a supportive partner, regular sexual activity, and better communication about sexual concerns, all of which can contribute to maintaining sexual satisfaction despite cancer-related challenges.

The contrasting variables associated with sexual functioning and satisfaction highlight the complex nature of sexual health. Whereas sexual functioning may be more influenced by physical and hormonal factors, such as treatment-induced menopause or surgical interventions, sexual satisfaction appears more closely tied to psychosocial elements such as relationship status and emotional well-being. This distinction underscores the need for multifaceted interventions addressing both the physiological and psychological aspects of sexual health in YWBC. For instance, interventions aimed at improving sexual functioning might focus on managing physical symptoms and hormonal changes, whereas those targeting sexual satisfaction might emphasize relationship counseling, body image support, and mental health care. The finding that different factors predict these 2 aspects of sexual health also suggests that clinicians should assess both domains separately, because improvements in one area may not necessarily translate to improvements in the other.

Several limitations of this study are acknowledged. We cannot exclude the possibility of unmeasured confounding factors affecting our results, such as sexual orientation, use of antidepressants or other medications affecting sexuality, and natural aging during follow-up. Furthermore, the reliance on self-reported patient outcomes is susceptible to reporting biases. In addition, there are missing data and any departures from random missingness could have influenced estimates. Moreover, all participants were treated in Mexican health-care facilities, and findings from this cohort may not extrapolate to racial/ethnic groups with differing sociocultural attitudes and norms around sexuality. Last, the definition of sexual activity that was used focused on partnered activities, which may not capture the full spectrum of sexual experiences. Despite these caveats, this study reinforces that sexual health represents a ubiquitous and often under-recognized survivorship issue profoundly affecting QoL for YWBC. Our findings underscore the complex interplay of biological, psychological, and social factors influencing sexual health outcomes.

These results have important clinical implications. First, they highlight the need for routine screening and open communication about sexual health issues beginning at diagnosis and continuing throughout survivorship care. Second, the identification of distinct trajectory groups suggests that some patients may benefit from more intensive or tailored interventions. Third, our findings reinforce the importance of a multidisciplinary approach to addressing sexual health in YWBC. Interventions should address both the physiological aspects (eg, menopausal symptoms) and psychosocial factors (eg, body image, relationship issues) influencing sexual health. Fourth, the strong association between sexual function and overall QoL emphasizes that addressing sexual health should be considered an integral part of comprehensive cancer care, not an optional add-on. Fifth, focusing on Mexican women with BC provides insights into the unique experiences of this demographic within the broader Hispanic/Latina population.

In conclusion, this study provides key insights into the long-term sexual health trajectories of YWBC. The persistent and sometimes worsening nature of sexual problems in this group calls for greater attention to this aspect of survivorship care. Future research should focus on developing and testing culturally sensitive interventions tailored to the unique needs of YWBC, particularly those at risk for delayed-onset or worsening sexual problems.

## Data Availability

All data are available upon reasonable request to corresponding author.
